# Aqueous flare and inflammatory factors in macular edema with central retinal vein occlusion: a case series

**DOI:** 10.1186/1471-2415-13-78

**Published:** 2013-12-11

**Authors:** Hidetaka Noma, Tatsuya Mimura, Maria Tatsugawa, Katsunori Shimada

**Affiliations:** 1 Department of Ophthalmology, Yachiyo Medical Center, Tokyo Women’s Medical University, Chiba, Japan; 2 Department of Ophthalmology, Medical Center East, Tokyo Women’s Medical University, Tokyo, Japan; 3 Department of Ophthalmology, Kure Saiseikai Hospital, Hiroshima, Japan; 4 Department of Biostatistics, STATZ Institute Inc., Tokyo, Japan

## Abstract

**Background:**

The association of inflammatory factors and the aqueous flare value with macular edema in central retinal vein occlusion (CRVO) patients remains unclear. We investigated the relations between the aqueous flare value and vitreous levels of vascular endothelial growth factor (VEGF), soluble intercellular adhesion molecule-1 (sICAM-1), and interleukin-6 (IL-6) in patients with CRVO and macular edema or patients with idiopathic macular hole (MH).

**Methods:**

In 38 patients who underwent unilateral vitrectomy (21 CRVO patients and 17 MH patients), vitreous samples were obtained during vitrectomy to measure VEGF, sICAM-1, and IL-6. Retinal ischemia was evaluated from capillary non-perfusion on fluorescein angiography, and the CRVO patients were classified into nonischemic or ischemic groups. Aqueous flare values were measured with a laser flare meter and macular edema was examined by optical coherence tomography.

**Results:**

The median aqueous flare value increased significantly across the three groups (MH group < nonischemic CRVO group < ischemic CRVO group). There was a significant correlation between the flare value and vitreous levels of VEGF, sICAM-1, and IL-6 in the CRVO group. The flare value was also significantly correlated with the severity of macular edema in the CRVO group.

**Conclusions:**

Inflammation and/or ischemia may increase vascular permeability and disrupt the blood-aqueous barrier by increasing levels of inflammatory factors in patients with CRVO and macular edema.

## Background

Central retinal vein occlusion (CRVO) is frequently associated with macular edema, which is the chief cause of visual impairment in these patients. Intravitreal injection of antibodies targeting vascular endothelial growth factor (VEGF), such as bevacizumab or ranibizumab, has been reported to improve macular edema in patients with CRVO [1,2]. However, some patients have persistent macular edema despite treatment with these antibodies [3,4], suggesting that factors other than VEGF may also contribute to macular edema. We recently reported that vitreous fluid levels of inflammatory factors were significantly correlated with the severity of macular edema in CRVO patients [5,6], suggesting that inflammation is important in the development of macular edema. This is supported by the Standard Care vs Corticosteroid for Retinal Vein Occlusion (SCORE) study, which showed that intravitreal triamcinolone acetonide improved visual acuity and macular edema in CRVO patients [7]. Moreover, vitreous fluid levels of inflammatory factors are reported to be high in ischemic CRVO [5,6], although some patients with nonischemic CRVO have increased levels of inflammatory factors, suggesting that inflammation may also promote macular edema in nonischemic CRVO.

The aqueous flare value is reported to be significantly higher in patients with retinal vein occlusion (RVO) than in normal controls [8,9], suggesting that an increased flare value reflects disruption of the blood-retinal barrier and blood-aqueous barrier by inflammation. However, the association of inflammatory factors and the aqueous flare value with macular edema in CRVO patients remains unclear. Accordingly, we investigated the relation between macular edema and inflammatory parameters (flare value and vitreous fluid levels of VEGF, sICAM-1, and IL-6) in patients with CRVO.

## Methods

### Subjects

This study was performed in accordance with the Helsinki Declaration of 1975 (1983 revision). The institutional review boards of Tokyo Women’s Medical University approved the protocol for collection and testing of vitreous fluid samples. Undiluted vitreous fluid samples were harvested at the start of vitrectomy after written informed consent was obtained from each subject following an explanation of the purpose and potential adverse effects of the procedure. This was a retrospective case control study of 38 Japanese patients who underwent vitrectomy in one eye (21 with CRVO and 17 with idiopathic macular hole (MH) to treat macular edema. Consecutive patients with CRVO who presented to the hospital of Tokyo Women’s Medical University between June 2007 and March 2011 were screened according to the criteria set out below and vitreous fluid samples were obtained from the 21 patients who were enrolled. The indication for pars plana vitrectomy was relief of vitreomacular traction in order to improve macular edema caused by CRVO. The inclusion criteria were (1) patients scheduled for pars plana vitrectomy to treat macular edema secondary to CRVO (including patients who had received retinal photocoagulation) and (2) patients with a best-corrected visual acuity of worse than 20/50 before surgery. Exclusion criteria were (1) previous ocular surgery or intravitreous injection of anti-VEGF agents or triamcinolone acetonide, (2) diabetes mellitus with diabetic retinopathy, (3) iris rubeosis, and (4) a history of ocular inflammation or vitreoretinal disease. Vitreous fluid samples were also obtained from 17 patients with nonischemic ocular disease (MH) as a control group (MH group). None of the patients in the MH group had proliferative vitreoretinopathy. The CRVO group (10 women and 11 men) was aged 71.2 ± 6.9 years (mean ± SD), while the MH group (10 women and 7 men) was aged 68.1 ± 4.9 years (Table 1). The mean duration of CRVO was 5.8 ± 2.8 months (range: 2 – 12 months) (Table 1). This report covers our recent work on the relationship between cytokines and the pathogenesis of CRVO. Therefore, the present study population partially overlaps subjects reported in previous papers [5,6,10]. Pars plana vitrectomy was performed at Tokyo Women’s Medical University. A diagnosis of hypertension or hyperlipidemia was based on data from the medical records. Before surgery, panretinal photocoagulation was done in 4 patients (4 eyes) to prevent neovascular glaucoma (mean: 1,039 shots; range: 790 to 1,624 shots). Hypertension was defined as current treatment with antihypertensive drugs or a blood pressure > 140/90 mmHg [11]. Hyperlipidemia was defined as current treatment with lipid-lowering agents or a total cholesterol level > 240 mg/dl. Twelve of the 21 CRVO patients (57%) had hypertension (Table 1). Seven of the 21 CRVO patients (33%) had hyperlipidemia (Table 1).

**Table 1:** Clinical and laboratory characteristics of the CRVO patients and MH patients

	**CRVO group**	**MH group**	***P *** **value**
Number^†^	21	17	
Gender (Female/male)	10/11	10/7	0.718
Age (yr)	71.2 ± 6.9^‡^	68.1 ± 4.9^‡^	0.128
Blood pressure (mmHg)			
Systolic	139 ± 18^‡^	116 ± 13^‡^	<0.001
Diastolic	78 ± 9^‡^	72 ± 8^‡^	0.058
Hypertension	12	2	0.004
Hyperlipidemia	7	3	0.275
Duration of CRVO (months)	5.8 ± 2.8^‡^	-	-

^†^Number of patients with data.

^‡^Mean ± standard deviation (SD).

*CRVO* = central retinal vein occlusion; MH = macular hole.

### Fundus examination

The fundus was examined preoperatively by biomicroscopy with a fundus contact lens and by standard fundus color photography. In addition, fluorescein angiography was performed with a Topcon TRC-50EX fundus camera, an image-net system (Tokyo Optical Co. Ltd., Japan), and a preset lens with a slit-lamp.

Preoperative fundus findings were recorded for each subject. A masked grader independently assessed retinal perfusion status or ischemic retinal vascular occlusion by examination of fluorescein angiograms. The ischemic area of the retina was measured with the public domain Scion Image program, as reported previously [5,6]. On a digital fundus photograph, the disk area was outlined with a cursor and then measured, as was the non-perfused area. If the nonperfused area divided by the disc area gave a value of 10 or more, this was defined as indicating the presence of retinal ischemia [12-14]. Sites of retinal photocoagulation were excluded when calculating the nonperfused area. The 21 CRVO patients included 15 patients with retinal ischemia (87.7 ± 26.6 disc areas), who were 9 women and 6 men aged 71.6 ± 6.9 years. The other 6 patients without retinal ischemia (3.8 ± 3.2 disc areas) comprised 1 woman and 5 men aged 70.3 ± 7.3 years.

Optical coherence tomography (OCT) was performed in each subject within 1 week before vitrectomy, employing an instrument from Zeiss-Humphrey Ophthalmic Systems (Stratus model 3000; Carl Zeiss, Dublin, CA, USA). The fundus was scanned with the measuring beam focused on horizontal and vertical planes crossing the center of the fovea, which was located by examination of the fundus photograph and by each patient’s fixation. Cross-sectional images were collected by a single experienced examiner, who continued each examination until highly reproducible scans were obtained. The thickness of the central fovea was defined as the distance between the inner limiting membrane and the retinal pigment epithelium (including any serous retinal detachment), and was automatically measured by computer software. The thickness of the neurosensory retina was defined as the distance between the inner and outer neurosensory retinal surfaces [15], and the severity of macular edema was graded from the measured retinal thickness. The average preoperative retinal thickness was 670 ± 155 μm, with a range of 434 to 976 μm.

### Measurement of aqueous flare

The aqueous flare was measured with a laser flare meter (FC-600, Kowa Co. Ltd., Tokyo, Japan), as described previously [16]. The sensitivity and reproducibility of this method have been confirmed by a number of studies [8,9,16]. Measurements were performed within 1 week before treatment. Flare values and cell counts were measured at 30 minutes after dilation of the pupil with 0.5% tropicamide and 5% phenylephrine hydrochloride. Two different examiners obtained five measurements from each eye and the results were averaged after excluding all measurements with artefacts.

### Sample collection

Samples of undiluted vitreous fluid (500 – 1,000 μl) were collected at the start of vitrectomy by aspiration into a 1 ml syringe attached to the vitreous cutter before commencing the intravitreal infusion of balanced salt solution. The vitreous samples were immediately transferred into sterile tubes and were rapidly frozen at -80°C.

### Measurement of inflammatory factors

The levels of VEGF, sICAM-1, and IL-6 were measured in vitreous samples from the same eye and in plasma samples by enzyme-linked immunosorbent assay using kits for human VEGF, sICAM-1, and IL-6 (VEGF and IL-6: R&D Systems, Minneapolis, MN; sICAM-1: Bender Med Systems, Burlingame, CA, USA) [5,6]. The VEGF kit detected two of the four VEGF isoforms, which were VEGF_121_ and VEGF_165_. The levels of these factors in the vitreous fluid samples and plasma were within the detection ranges of the assays, with the minimum detectable concentration being 15.6 pg/ml for VEGF (intra-assay CV 5.5%, inter-assay CV 6.7%), 3.3 ng/ml for sICAM-1 (intra-assay CV 5.4%, inter-assay CV 7.7%), and 0.156 pg/ml for IL-6 (intra-assay CV 5.5%, inter-assay CV 6.8%).

### Statistical analysis

All analyses were performed with SAS System 9.3 software (SAS Institute Inc., Cary, North Carolina, USA). Data are presented as the mean ± SD or as the median with the interquartile range or frequency. Student’s *t*-test was employed to compare normally distributed unpaired continuous variables between the two groups and the Mann-Whitney U test was used for variables with a skewed distribution. Probability values for trends among the three groups were calculated with a linear regression model. To examine the relations among vitreous levels of VEGF, sICAM-1, or IL-6, the aqueous flare value, and the severity of macular edema, Spearman’s rank-order correlation coefficients and a multiple linear regression model were used. Two-tailed p values of less than 0.05 were considered to indicate a statistically significant difference.

## Results

The aqueous flare value (median [interquartile range]) showed a significant increase across the three groups (MH group: 5.3 photon counts/ms [4.3-6.2] < nonischemic CRVO group: 9.3 photon counts/ms [8.7-10.5] < ischemic CRVO group: 25.1 photon counts/ms [20.7-34.6]) (*P*_*trend*_ < 0.001; Table 2). The vitreous fluid level of VEGF also showed a significant increase across the three groups (MH group: 15.6 pg/ml [15.6-15.6] < nonischemic CRVO group: 83.3 pg/ml [15.6-143] < ischemic CRVO group: 1050 pg/ml [335-3717]) (*P*_*trend*_ < 0.001; Table 2). Likewise, the vitreous fluid level of sICAM-1 showed a significant increase across the three groups (MH group: 4.1 ng/ml [3.6-5.8] < nonischemic CRVO group: 8.4 ng/ml [6.2-9.4] < ischemic CRVO group: 19.8 ng/ml [12.7-37.0]) (*P*_*trend*_ < 0.001; Table 2). Moreover, the vitreous fluid level of IL-6 showed a significant increase across the three groups (MH group: 1.06 ng/ml [0.85-1.52] < nonischemic CRVO group: 11.5 ng/ml [8.0-42.8] < ischemic CRVO group: 51.2 ng/ml [22.2-104.8]) (*P*_*trend*_ < 0.001; Table 2).

**Table 2:** Aqueous flare and vitreous levels of various factors in the control, nonischemic CRVO, and ischemic CRVO groups

**Variable**	**Control**	**Nonischemic**	**Ischemic**	***P *** **value for trend test**
Aqueous flare value	5.3 [4.3-6.2]	9.3 [8.3-10.5]	25.1 [20.7-34.6]	<0.001
(photon counts/ms)				
VEGF (pg/ml)	15.6 [15.6-15.6]	83.3 [15.6-143]	1050 [335-3717]	<0.001
sICAM-1 (ng/ml)	4.1 [3.6-5.8]	8.4 [6.2-9.4]	19.8 [12.7-37.0]	<0.001
IL-6 (pg/ml)	1.06 [0.85-1.52]	11.5 [8.0-42.8]	51.2 [22.2-104.8]	<0.001

*CRVO* = central retinal vein occlusion; *VEGF* = vascular endothelial growth factor; *sICAM-1* = soluble intercellular adhesion molecule 1; *IL-6* = interleukin-6.

There was a significant correlation between the aqueous flare value and the vitreous fluid levels of VEGF, sICAM-1, and IL-6 in the CRVO group (ρ = 0.73, *P* = 0.001, ρ = 0.66, *P* = 0.003, and ρ = 0.63, *P* = 0.005, respectively) (Table 3). Furthermore, to clarify which factor (VEGF, sICAM1, or IL6) was most closely correlated with the aqueous flare value, multiple linear regression analysis with stepwise selection of variables was performed. This analysis showed that sICAM-1 was most strongly correlated with the aqueous flare value. The aqueous flare value was also significantly correlated with the severity of macular edema in the CRVO group (ρ = 0.54, *P* = 0.015) (Table 3).

**Table 3:** Correlation of aqueous flare values with levels of vitreous factors and retinal thickness

	**ρ**	**P value**
VEGF (pg/ml)	0.73	0.001
sICAM-1 (ng/ml)	0.66	0.003
IL-6 (pg/ml)	0.63	0.005
Macular retinal thickness (μm)	0.54	0.015

VEGF = vascular endothelial growth factor; sICAM-1 = soluble intercellular adhesion molecule 1; IL-6 = interleukin-6; γ = ρ = correlation coefficient.

**Figure 1:**
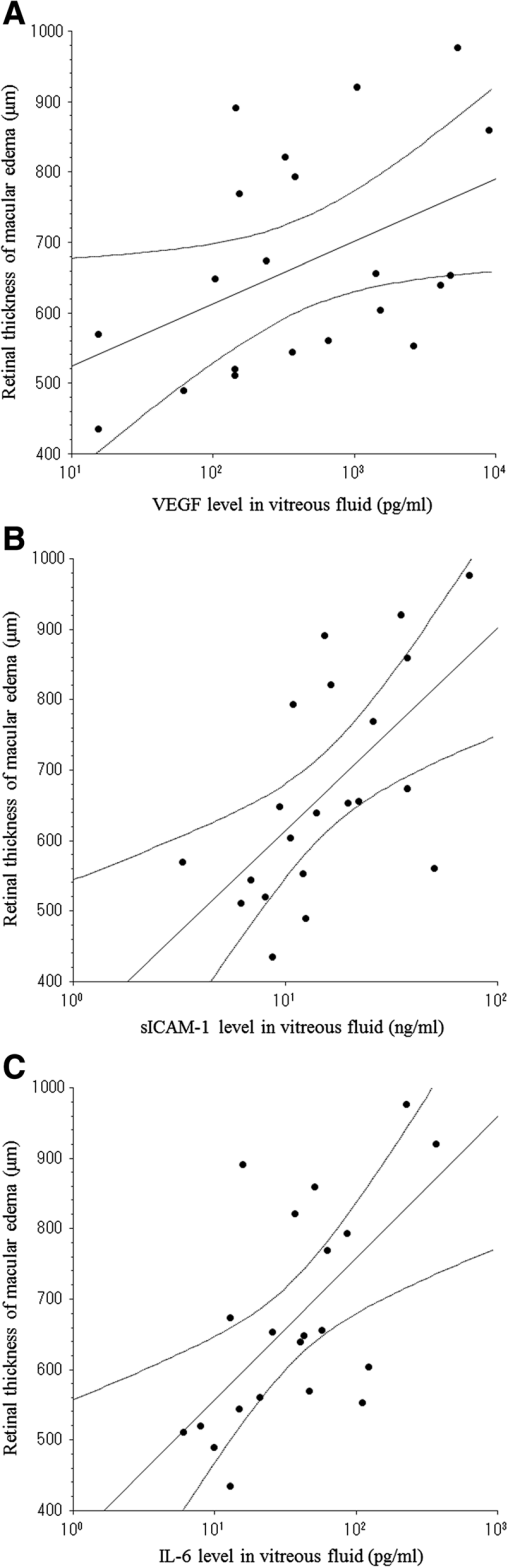
**Correlation between the severity of macular edema and the vitreous fluid levels of vascular endothelial growth factor (VEGF), soluble intercellular adhesion molecule-1 (sICAM-1), and interleukin-6 (IL-6).** In the CRVO group, vitreous levels of VEGF **(A)**, sICAM-1 **(B)**, and IL-6 **(C)** were significantly correlated with the severity of macular edema (ρ = 0.49, *P* = 0.029, ρ = 0.66, *P* = 0.003, and ρ = 0.61, *P* = 0.006, respectively).

Vitreous fluid levels of VEGF, sICAM-1, and IL-6 were also significantly correlated with the severity of macular edema in the CRVO group (ρ = 0.49, *P* = 0.029, ρ = 0.66, *P* = 0.003, and ρ = 0.61, *P* = 0.006, respectively) (Figures 1A-C). In addition, to clarify which factor (VEGF, sICAM1, or IL6) was most closely correlated with the severity of macular edema, multiple linear regression analysis with stepwise selection of variables was performed. It was found that sICAM-1 was most strongly correlated with the severity of macular edema.

In the CRVO group, there were no significant correlations between the aqueous flare value and age, hypertension, hyperlipidemia, or the duration of CRVO (data not shown, *P* = 0.680, *P* = 0.274, *P* = 0.918, and *P* = 0.450, respectively). There were also no significant correlations between the vitreous fluid level of VEGF and these variables (data not shown, *P* = 0.593, *P* = 0.413, *P* = 0.575, and *P* = 0.068, respectively). Likewise, there were no significant correlations between the vitreous fluid level of sICAM-1 and these variables (data not shown, *P* = 0.572, *P* = 0.644, *P* = 0.073, and *P* = 0.090, respectively), as well as no significant correlations between the vitreous fluid level of IL-6 and these variables (data not shown, *P* = 0.819, *P* = 0.831, *P* = 0.550, and *P* = 0.312, respectively).

## Discussion

The present study revealed that the protein concentration in aqueous humor (aqueous flare value) was significantly higher in the CRVO group than in the MH group, as well as showing a significant difference between the ischemic and nonischemic CRVO groups. In addition, vitreous fluid levels of inflammatory factors (VEGF, sICAM-1, and IL-6) showed a significant increase across the three groups from the MH group to the ischemic CRVO group. Furthermore, there was a significant correlation between the aqueous flare value and vitreous fluid levels of these inflammatory factors in the CRVO group. Therefore, the increase of the flare value in CRVO may be related to increased production of inflammatory factors (such as VEGF, sICAM-1, and IL-6) due to inflammation and/or ischemia. Miyake et al. [8] performed anterior chamber and vitreous fluorophotometry, demonstrating increased fluorescein concentrations in the anterior chamber and the posterior vitreous of patients with RVO, whereas fluorescein levels in the middle vitreous were low or normal. They concluded that the increase of the aqueous flare mainly reflects disruption of the blood-aqueous barrier in RVO patients and that this barrier may be damaged in the anterior segment. Furthermore, Virdi et al. [17] found an increase of fluorescein in the aqueous humor and leakage from vessels of the iris on fluorescein angiography in patients who had major branch RVO or CRVO without any evident iridic abnormalities or rubeosis. Fluorescein leakage from the iridic vessels has also been identified in monkeys with experimental RVO before the onset of iridic neovascularisation [17]. These reports and our results suggest that the aqueous flare value may be increased by leakage of protein from the iridic vessels due to disruption of the blood-aqueous barrier by inflammatory factors such as VEGF, sICAM-1, and IL-6.

In the present study, we detected a significant correlation between the aqueous flare value and the severity of macular edema in our CRVO patients (Table 3). This finding is supported by the report that cystoid macular edema is associated with more severe blood-aqueous barrier disruption [18], and also suggests that macular edema in CRVO patients may be related to inflammation and/or ischemia. There was also significant correlation between the severity of macular edema and the vitreous fluid levels of VEGF, sICAM-1, and IL-6 in our CRVO patients. Therefore, the correlation between the aqueous flare value and the severity of macular edema in the CRVO group points to a role of inflammatory factors (VEGF, sICAM-1, and IL-6) in the development of macular edema by increasing vascular permeability and/or diapedesis of leucocytes. Thus, these findings suggest that inflammation and/or ischemia may promote vascular permeability and damage the blood-aqueous barrier by increasing inflammatory factors in CRVO patients with macular edema.

Recently, the SCORE study [7] and the CRUISE study [4] showed that intravitreal triamcinolone acetonide or ranibizumab could improve visual acuity and macular edema in CRVO patients. However, these studies did not assess VEGF and inflammatory molecules in the aqueous humor or vitreous fluid. Our findings suggest that it might be better to measure VEGF and other molecules before selecting a patient’s treatment. Such an approach is supported by the report that aqueous samples are useful for investigating the role of certain factors in various diseases and for pharmacokinetic/pharmacodynamic studies [19]. However, it is time-consuming and expensive to measure these molecules in the aqueous humor. The present study revealed that vitreous fluid levels of VEGF, sICAM-1, and IL-6 were significantly correlated with the aqueous flare value and with the severity of macular edema in CRVO patients, and that there was also a significant correlation between the flare value and the severity of macular edema. Accordingly, aqueous flare data could possibly be used to assess the contribution of inflammation to macular edema associated with CRVO. If a patient has a high aqueous flare value, not only anti-VEGF therapy but also intravitreal injection of triamcinolone acetonide could be considered. Triamcinolone acetonide may improve macular edema by decreasing retinal capillary permeability via changes of tight junctions [20], or it could inhibit the signaling cascade involving VEGF and its receptor that increases microvascular permeability [21,22]. Corticosteroids may also prevent the production of various inflammatory molecules [23-25] that promote leukocyte adhesion and breakdown of the blood-retinal barrier, thus increasing vascular permeability [26,27]. Taken together with such reports, the present findings suggest that inflammatory factors could be targeted to prevent an increase of vascular permeability in CRVO patients with macular edema, and measurement of the aqueous flare value may help to select the best treatment strategy for CRVO-associated macular edema. However, a randomized, prospective, clinical trial comparing anti-VEGF therapy with triamcinolone acetonide (and combined therapy) would be required to assess efficacy for macular edema associated with CRVO.

## Conclusions

We found a significantly higher aqueous flare value in patients with CRVO than in those with MH. There was also a significant correlation between the aqueous flare value and the severity of macular edema in CRVO, and vitreous fluid levels of VEGF, sICAM-1, and IL-6 were significantly correlated with both the aqueous flare value and the severity of macular edema in our CRVO patients. To the best of our knowledge (based on a Medline literature search), this is the first report about the association of inflammatory factors and the aqueous flare value with macular edema in CRVO patients. These findings suggest that inflammatory factors like VEGF, sICAM-1, and IL-6 increase vascular permeability and disrupt the blood-aqueous barrier in CRVO patients with macular edema.

## Competing interests

No conflicting relationship exists for any author.

## Authors’ contributions

HN was involved in the design and conduct of the study. Collection and management of the data were done by HN, and KS, while analysis and interpretation of the data were performed by HN, TM, MT, and KS. Preparation of the first draft of the manuscript was done by HN, and review and approval of the manuscript was performed by TM, and KS. All authors read and approved the final manuscript.

## Pre-publication history

The pre-publication history for this paper can be accessed here:

http://www.biomedcentral.com/1471-2415/13/78/prepub
